# Tailoring the secretome composition of mesenchymal stem cells to augment specific functions of epidermal regeneration: an *in vitro* diabetic model

**DOI:** 10.3389/fmedt.2023.1194314

**Published:** 2023-06-12

**Authors:** Jacob G. Hodge, Jennifer L. Robinson, Adam J. Mellott

**Affiliations:** ^1^ Bioengineering Graduate Program, University of Kansas, Lawrence, KS, United States; ^2^ Department of Plastic Surgery, University of Kansas Medical Center, Kansas City, KS, United States; ^3^ Department of Chemical and Petroleum Engineering, University of Kansas, Lawrence, KS, United States; ^4^ Department of Orthopaedics and Sports Medicine, University of Washington, Seattle, WA, United States; ^5^ Department of Mechanical Engineering, University of Washington, Seattle, WA, United States; ^6^ Institute for Stem Cell and Regenerative Medicine, University of Washington, Seattle, WA, United States; ^7^ Ronawk Inc., Olathe, KS, United States

**Keywords:** extracellular vesicles, hydrogels, regenerative medicine, secretome, stem cells, wound healing

## Abstract

**Introduction:**

Wound healing consists of a dynamic series of events that are highly dependent on paracrine factors for proper progression through the phases of wound healing. Inappropriate progression through the phases is associated with insufficient epidermal regeneration (i.e., re-epithelialization) of wounds and subsequent propagation of chronic wounds, such as diabetic ulcers, which are associated with increased patient morbidity. Recently, investigation into the dynamic secretome of Adipose-derived Mesenchymal Stem Cells (ASCs), have shown promise in augmenting the wound healing response of chronic diabetic wounds. However, currently utilized 2D culture techniques are known to drastically alter the regenerative phenotype of ASCs. In this study a novel tissue-mimetic 3D system was utilized as a means to culture ASCs.

**Methods:**

The capacity for the ASC secretome to augment epidermal regeneration activity was then evaluated after exposure of ASCs to “wound priming stimuli” in 2D and 3D. The priming stimuli consisted of coating the 2D and 3D systems with the wound matrix proteins, collagen type I, fibronectin, and fibrin. To understand the potential benefit of the ASC secretome in the context of diabetic wounds, keratinocytes (KCs) were exposed to super-physiological glucose levels to induce a diabetic-like phenotype (idKCs).

**Results:**

Relative to KCs, idKC exhibited a 52% and 23% decline in proliferation and migration, respectively. Subsequently, analyses of the ASC secretome were performed. ASC conditioned media (ASC-CM) from tissue-mimetic culture demonstrated a > 50% increase secretion of proteins and a 2-fold increase in secreted EVs, relative to 2D culture. Interestingly, the different priming stimuli did not alter the total amount of protein or EVs secreted within the tissue-mimetic system. However, evaluation of specific soluble proteins via ELISA revealed significant differences in key epidermal regeneration factors, such as EGF, IGF-1, FGF-2, MMP-1, TIMP-1, and TGF*β*-1. Additionally, the relative effect of ASC-EVs from the 2D and 3D system on idKCs epidermal regeneration functionality varied significantly, with EVs from 3D-Collagen culture providing the most significant benefit on idKC activity.

**Discussion:**

Together, these data support the utilization of tissue-mimetic culture system to enhance the adaptability and secretory activity of MSC-like populations in order to generate tailored biologics, via priming stimuli, for specific wound healing applications.

## 1. Introduction

Wound repair and regeneration is a complex and dynamic series of events that occurs in the setting of tissue damage and involves a diverse array of cell populations (1, 2). For effective restoration of tissue function and anatomical homeostasis to occur, tightly controlled cellular and molecular signaling cascades promote migration and proliferation of cells, extracellular matrix deposition/remodeling, and cytoskeletal modulation of the local wound healing cell populations (1, 2). However, many patients and wound types can be predisposed to inadequate healing and protraction of the wound healing response that can ultimately result in progression towards a chronic wound phenotype (3).

The epidermal barrier of the skin is instrumental in maintaining the viability of deeper tissue structures and allowing proper wound healing processes to occur (4). Thus, a critical step of wound healing after an injury is the process of re-epithelialization (epidermal regeneration), which is carried out by epidermal keratinocytes with the goal of reestablishing the skin's external barrier properties (4, 5). Proper re-epithelialization requires keratinocytes to undergo a phenotypic switch. This phenotypic switch includes altering cytoskeletal and junctional proteins, such as cell-cell and cell-matrix junctions, to become more migratory and proliferative in order to repopulate and “close” the wound (6). Additionally, keratinocytes must alter their keratin expression from a more proliferative basal type (keratin 5 and 14) to expressing keratins associated with suprabasal differentiation (keratin 10) and an active wound response (keratin 16) (4). Feedback and paracrine signaling from surrounding cell populations help orchestrate proper re-epithelialization, including critical crosstalk between dermal fibroblasts, dermal adipose, and other epidermal keratinocytes (2). However, this cellular communication can become dysregulated due to imbalanced paracrine signaling and result in perpetually non-healing wounds that do not close, often progressing to significant limb disease (7, 8).

Chronic wounds are often a consequence of comorbid medical conditions like diabetes, where up to 15% of diabetics develop ulcerative wounds with a greater than 50% recurrence rate (9, 10). Diabetic wounds inherently have an improper balance and composition of bioactive compounds within the tissue, such as depleted growth factor bioavailability, imbalanced proteolytic activity, and a pro-inflammatory cytokine profile (9, 11). Moreover, high glucose levels are known to results in increased oxidative damage and glycation of cellular proteins (12–14). This propagates the abnormal progression of a number of wound healing processes, including proper keratinocyte differentiation and functional activity associated with epidermal regeneration. Notably, keratinocytes isolated from diabetic wounds have been shown to also lack the same capacity to alter their keratin expressional pattern and properly migrate (4, 11). Thus, diabetic wounds are associated with a cycle of sustained, inappropriate cellular signaling and inadequate neotissue formation (11). Consequently, lack of wound closure can result in polymicrobial infections, desiccation, and reinjury of diabetic wounds, which remain the leading cause of non-traumatic lower limb amputations with an associated 5-year mortality post-amputation ranging from 55%–70%, second only to lung cancer (15, 16). The principle of decreased wound healing capabilities of diabetic keratinocytes was taken advantage of within this study via generation of an inducible diabetic-like keratinocyte population through sustained exposure to high glucose levels in order to generate functional deficits in keratinocytes.

The harsh and complex nature of chronic wounds and their tissue microenvironments has also resulted in a lack of development of focused and effective therapeutic interventions, with inadequate re-epithelialization remaining a major limitation of current chronic wound therapies (7, 8). Therefore, there remains a critical need to develop a therapy that can help circumvent the loss of proper keratinocyte functionality and restore the appropriate re-epithelialization capacity of wounds to prevent/revert progression of complex chronic wounds. Bioactive compounds, such as growth factors (GFs) and extracellular vesicles (EVs), coordinate tissue reparative processes and hold immense regenerative capabilities (17, 18). Yet, enhanced proteolytic degradation, radial diffusion, and a lack of biological diversity from single GF administration in prior clinical trials has led to mixed results of therapies aimed at utilizing GFs for chronic wounds (19–22). However, the diverse yet balanced milieu of GFs, antioxidants, extracellular vesicles (EVs), immunomodulatory cytokines, proteases and proteolytic inhibitors secreted from adipose-derived mesenchymal stem cells (ASCs) could offer a unique approach to develop targeted therapies for treating chronic wounds that circumvent the limitations of single GF and/or autologous cell therapies (23–25).

Currently, the most common therapeutic modalities used clinically for chronic wound care are moist dressings and continuous surgical debridement (8, 26, 27). However, early initial studies with ASCs in *in vivo* animal models and clinical trials have shown the ability for ASCs to potentially enhance the rate of wound “closure” and improve diabetic wound outcomes (28–30). Recent studies have demonstrated that a key component of the inherent regenerative capabilities of ASCs is their secretome (31–33). ASC populations and their secretome are highly diverse and adaptable, allowing them to rapidly respond to a variety of environmental stimuli in a state-dependent manner, such as within a wound environment. Notably, both direct injection of ASCs and ASC secretory compounds into chronic wounds have previously demonstrated improved outcomes *in vivo* (30, 34). Interestingly, hypoxia priming of ASCs has been shown to upregulate secretion of angiogenic compounds and priming with inflammatory cytokines has been shown to upregulate secretion of anti-inflammatory and mitogenic compounds from ASCs (35). Moreover, fibrin, a matrix compound and native wound healing stimulus, has been used as a delivery vehicle for ASCs and demonstrated the ability to improve overall wound outcomes, including in chronic wounds (36). Additional matrix-derived biomaterials have also previously been investigated to control the bioactivity of MSC populations, including collagen I and fibronectin, which are both intricately involved within native wound tissue, and have demonstrated the ability to promote wound healing (37, 38). Thus, ASCs demonstrate an inherent capacity to adaptively respond to environmental stimuli via modulating their secretory activity in a state-dependent manner, including exposure to wound microenvironment-derived stimuli (e.g., hypoxia, inflammation, and matrix compounds).

While previous data with MSC-derived biologics and cell therapies have demonstrated promise, to date, they have almost exclusively utilized 2D culture expansion to achieve adequate quantities of biological products (e.g., cells or secretome). Notably, the biochemical and biomechanical cues that cells are exposed to within the native tissue microenvironment are key to regulating cellular phenotype and activity, especially in mesenchymal stem cell (MSC) populations such as ASCs (39, 40). Thus, culture of ASCs within an unphysiological and rigid 2D culture system has been shown to promote a significant decline in ASC phenotype and viability (41, 42). Consequently, 2D cultured ASCs lose their inherent adaptability and regenerative properties, leading to inconsistent and tainted secretory products (43). However, culture of ASCs within softer 3D systems that more closely resemble native tissue mechanics have been shown to retain the native ASC phenotype longer and result in secretion of more robust regenerative compounds (44–47). Thus, recent investigations have looked to develop 3D tissue-mimetic systems that enhance culture of ASC populations and permit easily tailorable properties to modulate ASC phenotypic activity (e.g., introduction of exogenous priming stimuli). To date, most data on ASC priming involves 2D culture systems or 3D spheroids, and focus on enrichment of specific soluble factors secreted by ASCs (e.g., increased VEGF secretion), whereas data on understanding “functional” changes to the entire ASC secretome in the context of chronic wounds are still being investigated. Based on previous clinical trials and literature, we hypothesize that treatment with the complete and balanced ASC secretome will enhance keratinocyte function and epidermal regenerative activity due to the additive and synergistic effects of all secretory compounds (e.g., GFs, EVs, antioxidants, antiproteases), although certain compounds within the secretome may provide more potent stimuli in specific situations.

Due to the health status of patients with chronic (e.g., diabetic) wounds, the regenerative capacity of autologous ASCs often lacks the same ability to improve wound healing to the same extent as that seen with healthy ASCs (48, 49). Thus, ASC-derived acellular byproducts offer a cell-free alternative to current cell-based regenerative therapies and have shown immense paracrine effects, including the modulation of epidermal regeneration. In this study, a tissue-mimetic 3D hydrogel system, mechanically analogous to native adipose tissue, was utilized for culture of ASCs and compared/contrasted to traditional 2D culture (47, 50). Both 2D and 3D systems were coated with a variety of matrix-derived substrates native to wound tissue in order to assess the modulation of the ASC secretome for enhanced epidermal regeneration functional activity in keratinocytes. This study aims to provide valuable insight into additive and/or synergistic effects of priming allogeneic ASCs with different substrates while controlling for their mechanical environment via a tissue-mimetic 3D system. Thus, the more robust ASC populations within the same 3D mechanical environment, but exposed to different matrix compounds found within wounds, will potentially provide new insight into native signaling responses of ASCs to wound tissue; while also demonstrating the ability to produce tailored acellular biologics for specific applications.

## 2. Materials and methods

### 2.1. Cell culture

Human adipose-derived mesenchymal stem cells (ASCs; Lonza, Lot #18TL212639, 23-year-old Female, Black), human keratinocytes (KCs; Lonza, Lot #18TL318559, 62-year-old Male, Caucasian), were utilized in this study. ASCs were cultured in RoosterNourish MSC-XF (RoosterBio; Cat. #KT-016) for growth media (MSC-GM) and switched to RoosterCollect EV-Pro (RoosterBio; Cat. #K41001) for serum-free, low particulate media. DermaLife K Keratinocyte Medium Complete Kit was obtained from Lifeline Cell Technologies (Maryland, USA; #LL-0007) and used for KC culture. Traditional T-150 flasks were used for expansion until ∼80% confluency was reached and subculturing (i.e., passaging) of ASCs and KCs was performed. A functional diabetic phenotype was induced in “Passage 1 (P1)” keratinocytes (KCs) via dosing KC-GM with a high dose (25 mM) of glucose for 10 days to achieve an “induced-diabetic” phenotype in keratinocytes (idKCs) (51, 52). An initial characterization of P1 ASC phenotype was performed, and P2 ASCs were utilized for this study in its entirety to eliminate any effect of subculturing. 2D and 3D cells were seeded at the same seeding density of ∼1,500 cells/cm^2^ and 200 µl media/cm^2^ to allow for analogous comparison between 2D and 3D culture and conditioned media. The 3D hydrogel system is ∼1-cm^3^ and is a 3D-printed cell culture and expansion system called an X-Block (Ronawk; Kansas, USA) that contains a unique microarchitectural design that permits mass transport and nutrient exchange. The tissue-mimetic 3D hydrogels were placed into a glass 6-well culture plate for culturing of ASCs. Coating of the 2D and 3D systems was performed 24-h before cell seeding. Cells were then added dropwise to the surface of the hydrogels and allowed to migrate/disperse into the porous microarchitecture. Coating substrates included a thin coating of either collagen type 1 (Corning; Cat. #354265) at a concentration of 1-µg/ml, fibronectin (Corning; Cat. #356008) at a concentration of 1-µg/ml, or fibrin at a concentration of 10-µg/ml. Fibrin was fabricated vi*a* coating with 10-µg/ml of fibrinogen (Sigma; Cat. #F3879) for 24 h, followed by 3× washes in HBSS and addition of 10 units of thrombin (Sigma; Cat. #T6884) for 30 min. A non-coated control was used for both 2D and 3D. All samples were then gently submerged in HBSS 3× to wash any residual substrate.

### 2.2. Assessment of ASC phenotype

Initial assessment of adipogenic, chondrogenic, and osteogenic trilineage differentiation potential of ASCs (at P1) was performed via culture with differentiating media, according to the manufacturer's instructions. Adipogenic differentiation was performed using hMSC Adipogenic Differentiation BulletKit™ (Lonza; Cat. #PT-3004) and assessed for adipogenesis v*ia* Oil Red O assay (ScienCell; Cat. #0843). Chondrogenic differentiation was performed using hMSC Chondrogenic Differentiation Medium BulletKit™ (Lonza; Cat. #PT-3003), and was supplemented with TGF-β3 (Lonza; PT-4124) at a concentration of 10-ng/mL and assessed for chondrogenesis via Alcian Blue assay (ScienCell; Cat. #8378). Osteogenic differentiation was performed using hMSC Osteogenic Differentiation Medium BulletKit™ (Lonza Cat. #PT-3002) and assessed for osteogenesis via Alizarin Red S assay (ScienCell; Cat. #0223). Evaluation of ASC “stem-like” phenotype was also evaluated at P1 for positive immunolabeling for CD73/90/105, and negative immunolabeling for CD34/45. All primary antibodies were obtained from Abcam (Cambridge, UK) unless otherwise stated. In brief, ASCs were seeded in 2D for 24 h, fixed in 4% paraformaldehyde for 15-min, washed 3× with HBSS, blocked with 2% donkey-serum, and immunolabeled for CD34 (ab81289), CD45 (ab40763), CD90 (ab181469), CD105 (ab231774). CD73 (Cat. #41-0200) was obtained from Invitrogen (Waltham, MA). Hoechst 33342 (Invitrogen; Cat. #H3570) and Alexa Fluor 488 Phalloidin (ThermoFisher; Cat. #A12379) were used as counterstains. ASC were cultured in 2D or 3D systems for 8 days and then were extracted for proteomic and phenotypic analyses. For ASC cell number quantification, upon extraction, cells were centrifuged to pellet the cells, lysed with 0.5% Triton-X and evaluated via PicoGreen Quant-iT™ dsDNA Assay Kit (Invitrogen; Cat. #P7589), per the manufacturer's instruction.

### 2.3. Isolation of ASC conditioned media

ASC conditioned medium (ASC-CM) collection was performed via MSC-GM media removal, cells were then washed 3× with HBSS and serum-free MSC media was added for an additional 24-h wash. The 24-h wash was removed and new serum-free media was added followed by collections at 24-h intervals for three consecutive days during days 6–8 of culture. The ratio of media/cell was standardized for all groups. Collected ASC-CM was centrifuged at 1,500 g for 10-min to eliminate cell debris, Steriflip filtered with a 0.22-μm filter, and stored at −80°C for long-term storage until use. ASC-CM from each day (6, 7 or 8) for each replicate (*n* = 3) was combined to create a “batch” mixture to achieve adequate volumes for all experiments and eliminate any potential variability between ASC-CM from different media collection days.

### 2.4. ASC-CM extracellular vesicle (Ev) production

EVs were isolated via ASC-CM centrifugation at 4,000 g for 30-min through a Vivaspin 20 MWCO 100,000 kDa centrifuge cutoff filter (Cytiva; Cat. # 28932363) followed by washing with PBS and re-centrifugation at 4,000 g for 5-min through the 100-kDa filter, for a total of 3 washes. EVs were precipitated from the remaining >100-kDa concentrate overnight using a ExoQuick-TC kit (SBI; Cat. # EXOTC10A-1), per the manufacturers protocol. The EV samples (*n* = 3) were resuspended in PBS and aliquots were removed and used to quantify relative protein content as an indirect measure of EV content via Pierce™ BCA Protein Assay Kit (Invitrogen; Cat. #23225), QuickDrop (Molecular Devices; SpectraMax QuickDrop Micro-Volume Spectrophotometer) quantification via absorbance at 280-nm, and Pierce™ Coomassie “Bradford” Protein Assay Kit (Invitrogen; Cat. # 23200). Purified EV samples were also evaluated via Nanoparticle Tracking Analysis (NTA; Malvern Panalytical; Nanosight LM10) to further assess particle concentrations and size distributions.

### 2.5. ELISAs of ASC-CM soluble protein

Twelve Quantikine Enzyme-linked immunosorbent assays (ELISAs) were purchased (R&D Systems) and used to quantify the concentration of twelve key proteinaceous factors within the ASC-CM that are involved in wound healing and epidermal regeneration. The twelve assays included Epidermal Growth Factor (EGF), Heparin-binding EGF (HB-EGF), Insulin-like Growth Factor (IGF-1), Fibroblast Growth Factor 2 (FGF-2), Keratinocyte Growth Factor (FGF-7), Transforming Growth Factor Beta 1 (TGF-β1), Interleukin 1 Beta (IL-1 β), Interleukin 1 Receptor Antagonist (IL-1Ra), Pro-Matrix Metalloprotease 1 (MMP-1), Matrix Metalloprotease 9 (MMP-9), Tissue Inhibitor of Metalloproteases 1 (TIMP-1), Tissue Inhibitor of Metalloproteases 2 (TIMP-2). Assays were carried out per the manufacturer's instructions and only performed on the soluble proteins within the previously collected ASC-CM. If protein concentration was below limit of detection for the ELISA on first attempt, ASC-CM protein was concentrated 10× and re-ran, but if still no protein was detected, then that protein of interest was deemed too low to quantify.

### 2.6. ASC-CM antioxidant composition

Collected ASC-CM sample antioxidant activity was assessed with a Total Antioxidant Capacity (TAC) Assay kit (Cell Biolabs; Cat. #STA-360), per the manufacturer's instructions. The TAC kit evaluates antioxidant activity via the reduction of copper (II) to copper (I) and utilizes the naturally occurring antioxidant uric acid as a control standard. Thus, antioxidant activity was displayed as “mM equivalents” of uric acid. Control serum-free MSC media was used to assess baseline antioxidant activity of media without exposure to cells. Assays were performed with technical replicates and biological triplicates (*n* = 3).

### 2.7. KC and idKC functional activity after ASC-CM treatment

KCs and idKCs were plated in 2D cultures plates and allowed to acclimate and achieve appropriate confluences (>24 h) for each assay. KC-GM was then removed, cells were washed, ASC-CM was applied for 24-h, and experimental assays for metabolic, mitochondrial, proliferative, or migratory activity were then performed per the manufacturer's instructions. ASC-CM was used as a “supplement” for the Keratinocyte Growth Media (KC-GM) and dosed at a 2:1 ratio (ASC-CM to KC-GM). In short, plated cells were analyzed via PicoGreen fluorescence obtained at 435/535 nm (*n* = 3) to quantify DNA as a surrogate measurement of proliferation. PrestoBlue fluorescence was obtained at 560/590 nm (*n* = 3) and displayed as an average relative fluorescent unit (R.F.U.) of PrestoBlue per Hoechst signal (350/460 nm) to obtain approximate metabolic activity per cell. KC scratch assays were performed to evaluate changes in wound size/area as a surrogate measurement of KC migration after inflicted a scratch within a confluent monolayer of KCs (*n* = 3). Migration images were taken using an ImageXpress Micro XLS Imaging System (Molecular Devices) and the percent of wound area closed at 24-h was determined via ImageJ analysis. Subsequently, EVs isolated from both 2D and 3D coated and non-coated samples, were added to KC-GM at a final concentration of 150 μg/ml EV protein. Concentration of EVs was determined via previously performed protein quantification, and idKCs were then evaluated for phenotypic and functional changes in epidermal regeneration capacity (metabolic, proliferation, migration) to assess variability in quality/composition of EVs from each group.

### 2.8. Quantitative real-time PCR (qRT-PCR) expression analysis

RNA was isolated and purified via an RNeasy Mini Kit (Qiagen). Only RNA with a 260/280 ratio of >1.8 were used for this study. Cycle threshold (Ct) values were recorded and analyzed via the Delta-Delta-Ct method. qRT-PCR analysis was performed on ASCs via a wound healing array and on KCs via individually selected primers. For ASCs, both 2D and 3D cultured ASCs (with or without coatings) were collected during the experimental assays after ASC-CM collections and were assessed via an RT² Profiler™ PCR Array for Human Wound Healing (Qiagen; Cat. #330231; PAHS-121ZC-24) to evaluate expression of 84 wound healing and wound healing-associated genes. Glyceraldehyde 3-phosphate dehydrogenase (GAPDH), Beta-actin (ACTB), and Beta-2-Microglobulin (B2M) were the endogenous control genes utilized by the array. For KCs, untreated KCs and idKCs cultured in KC-GM served as a control for each group. Purity of cDNA samples was assessed with a QuickDrop spectrophotometer (Molecular Devices), with a 260/280 absorbance ratio >1.8 was designated as pure. Individual qPCR primers (Qiagen; Cat. #330001) were purchased to perform RT-qPCR on CDKN2A, CDH1, CDH2, FLG, KRT5, KRT10, KRT16, TWIST1, EGFR, VIM, TWIST1, IL1B, and CCND1. Expression of GAPDH was used as an endogenous control for all samples. All qRT-PCR samples were performed in triplicate (*n* = 3).

### 2.9. Statistical analysis

All data were reported as means with standard deviation. Comparative functional analyses of initial induced diabetic KCs were evaluated with a One-way ANOVA. All remaining statistics, including ASC-CM assays with keratinocytes, ASC-CM protein, EV quantification, ELISAs, and RNA analyses were evaluated with a Two-way ANOVA. A minimum of three biological replicates (*n* = 3) was used unless otherwise stated. Data were tested for normality via Shapiro-Wilk and Kolmogorov-Smirnov tests and plotted with a QQ plot. GraphPad Prism 9.4.2 software (La Jolla, CA) was used for the analyses and a *p* < 0.05 was considered significant. ImageJ was utilized for image processing.

## 3. Results

### 3.1. Characterization of ASC phenotype and substrate coating

**Figure 1:**
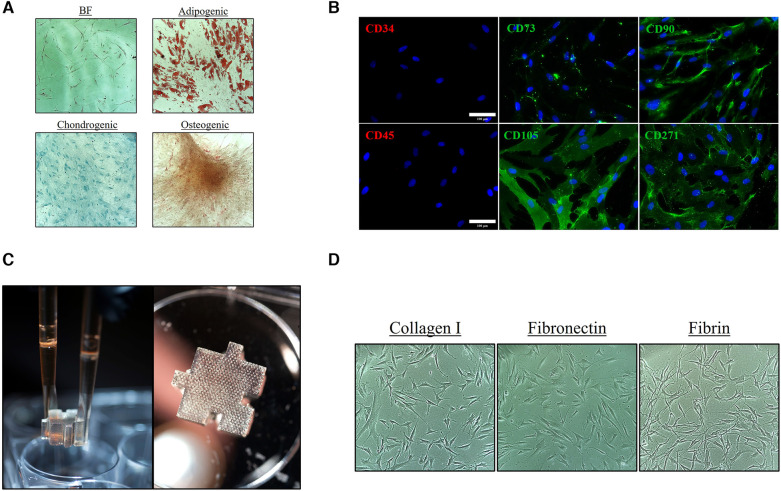
Characterization of ASC phenotype and substrate coating. Characterization of the initial ASC P1 population with (**A**) Spindle/Mesenchymal-like adherent cells with trilineage/multipotent potential and (**B**) positive CD surface marker expression of CD73/90/105/271 and negative expression of CD34/45 [Scale bar = 100 μm]. (**C**) Photographs of ∼1 cm^3^ tissue-mimetic X-Block inserted within a 6-well culture vessel. The textured appearance of the hydrogel is a result of the microporous architecture that traverses throughout the entire hydrogel. (**D**) ASCs were cultured on collagen type I, fibronectin, and fibrin coating 2D and 3D surface to assess for phenotypic and morphological changes.

To assess for an “MSC-like” phenotype, the ASCs were initially evaluated to determine that they were adherent (Figure 1A), exhibited trilineage multipotent potential (Figure 1A), and expressed MSC “stem-like” surface markers (Figure 1B), including positive staining for CD73/90/D105/CD271 and negative staining for CD34/45. Photographic images of the 3D hydrogel system were acquired to provide a visual of the macro-structure and the porous architecture (Figure 1C). The 2D and 3D systems were then coated with different matrix-derived proteins and ASCs were cultured for eight (8) days. Throughout culture the ASCs were imaged to observe morphological changes in 2D and 3D and with the different substrate coatings (Figure 1D and Supplementary Figure S6).

### 3.2. High glucose supplementation induces “functional” diabetic phenotype of KC populations

**Figure 2:**
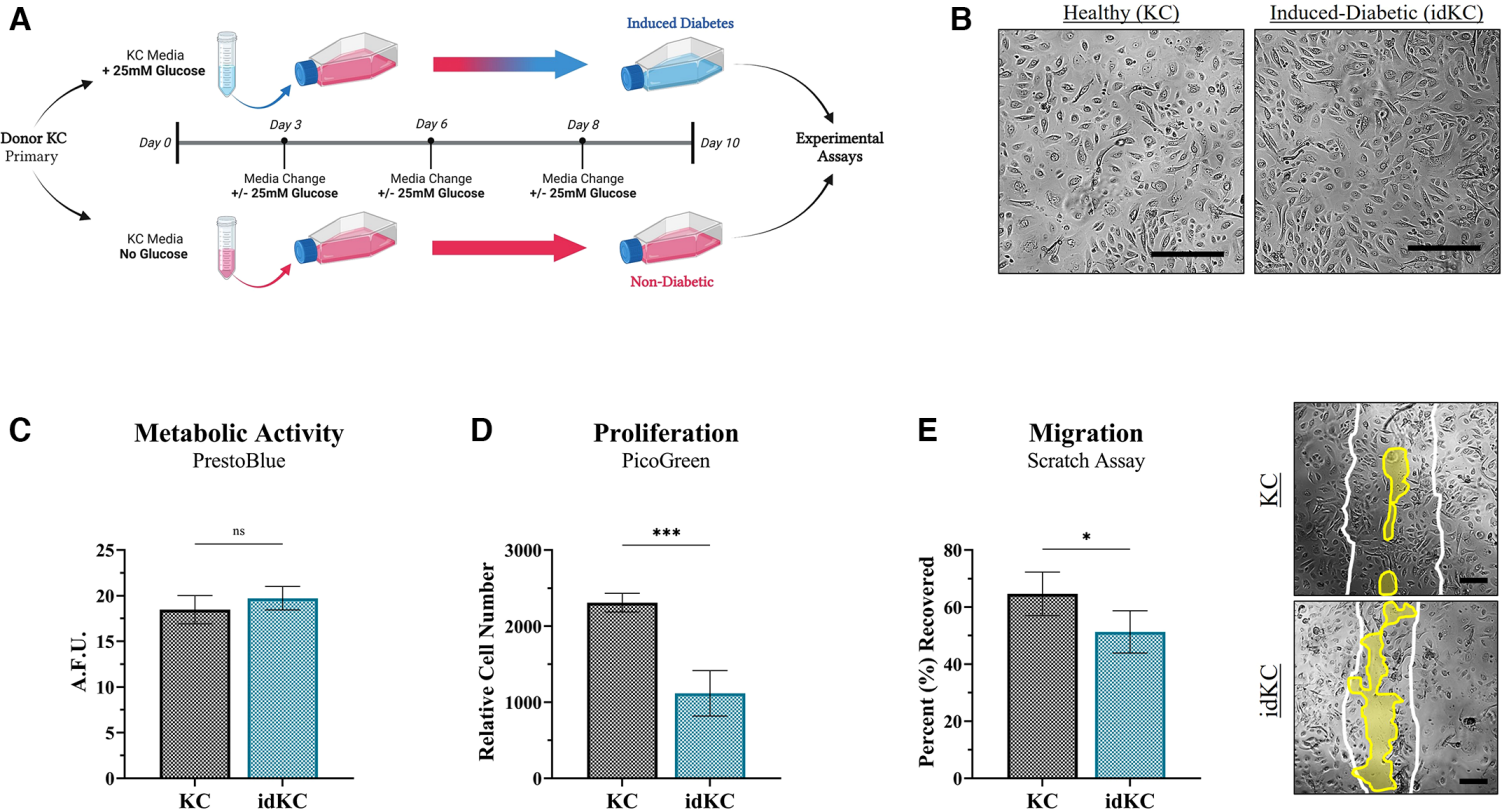
Induced-diabetic keratinocytes (idKCs) exhibit decreased epidermal regeneration activity. (**A**) Schematic diagram of the process of inducing a diabetic-like phenotype in KCs based on prior literature, performed in parallel to healthy KCs from the same donor to allow for direct comparison if KCs and idKCs from the same donor. KCs at “P1” were seeded in separate culture flasks, with 25 mM treatment resulting in induction of diabetes after 10 days. (**B**) Morphological images of healthy vs. idKCs. The idKCs population have an apparent shift towards more elongated-shaped cells [Scale bar = 100 μm]. The functional effect of diabetes induction on epidermal activity of idKCs (*teal bar*) relative to healthy control KCs (*black bar*) was evaluated via (**C**) metabolic, (**D**) proliferative, and (**E**) migratory changes in the idKC populations. A representative image of the scratch assay at 24 h is provided for KC and idKCs. White lines depict original “wound” edge. Yellow region highlights remaining region not recovered. [Scale bar = 50 μm]. Significance denoted as *p < 0.05 or ***p < 0.001.

To achieve a phenotype of KCs that functionally exhibited similar properties as native diabetic keratinocytes that exhibit decreased healing, a prolonged culture with super-physiological glucose levels was performed (Figure 2A). KC populations, with or without 25 mM glucose exposure, were then evaluated for changes in epidermal functional activity. Cell size of both healthy KCs and idKCs remained relatively similar, although idKCs exhibited a slight change in cell shape towards a more elongated/ellipsoid morphology, relative to the more rounded KC shape (Figure 2B). The metabolic activity (Figure 2C), proliferative activity (Figure 2D), and migratory activity (Figure 2E) were all assessed for healthy KC and idKCs. The metabolic activity of KCs and idKCs was similar; however, the proliferative and migratory activity of idKCs demonstrated significant declines within 24 h, with a ∼52% and 23% decline in proliferative and migratory activity, respectively.

### 3.3. Matrix substrates Alter ASC secretion of factors that modulate epidermal regeneration functional activity

**Figure 3:**
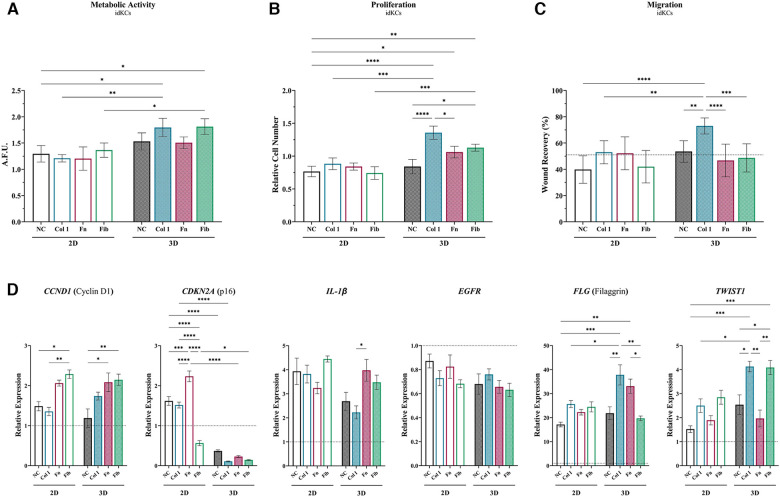
Matrix substrates alter ASC secretion of factors that modulate epidermal regeneration functional activity in idKCs. The effect of ASC-CM from 2D (*silhouette*) and 3D (*patterned*) systems that were coated (or non-coated control) was evaluated for ability to modulate idKC (**A**) metabolic, **(B**) proliferative, and (**C**) migratory activity. Functional activity data are denoted as relative change to baseline control idKCs, which were cultured with keratinocyte growth media (KC-GM). Migratory data depicted as percent (%) area recovered. Dashed line depicts idKCs control. (**D**) qRT-PCR analysis of *CCND1, CDKN2A, IL1B, EGFR, FLG*, and *TWIST1*. *GAPDH* was used as an internal control. Values are represented as relative fold change to baseline control idKC expression, indicated by dashed line, using the ΔΔCt method. NC, non-coated; Col 1, collagen type I; Fn, fibronectin; Fib, fibrin. Significance denoted as *p < 0.05, **p < 0.01, ***p < 0.001, and ****p < 0.0001.

Next, the additive and/or synergistic benefits of coating materials within the 3D hydrogel system, relative to 2D, was evaluated via assessing the effect of ASC-CM on the epidermal regeneration functionality of idKCs. ASC-CM from cells cultured within the coated tissue-mimetic system had an increased propensity for enhancing the metabolic activity of idKCs, relative to ASC-CM from any of the 2D coating groups, with only 3D-Col (1.79-fold) and 3D-Fib (1.81-fold) resulting in a significant increase in idKC metabolic activity relative to their 2D coated counterparts (Figure 3A). Interestingly, the non-coated 3D ASC-CM group maintained no difference in improving proliferative activity of idKCs relative to all 2D groups, whereas all coating in 3D significantly improved proliferative capacity of idKCs relative to traditional 2D-NC samples (Figure 3B). Moreover, ASC-CM from 3D-Col (1.36-fold) and 3D-Fib (1.13-fold) significantly increased idKC proliferative capacity relative to their 2D coated counterparts, 2D-Col (0.88-fold) and 2D-Fib (0.74 -fold) (Figure 3B). Similarly, no coatings in 2D had a significant effect on augmenting the ASC-CM to enhance idKC migratory activity, a key component of epidermal regeneration, whereas ASC-CM from 3D-Col resulted in the greatest increase in idKC migratory activity. Moreover, 3D-Col significantly enhanced idKC migratory activity relative to all other 3D ASC-CM groups and was the only ASC-CM group to significantly alter idKC migratory activity relative to the idKC control group (*received no ASC-CM dosing*), with ∼73% vs. ∼62% percent “wound” closure in 24 h, respectively (Figure 3C).

Further evaluation of idKC activity was performed via assessment of gene expression for markers associated with epidermal regeneration activity (Figure 3D). Cyclin D1 (*CCND1*) was evaluated to assess for changes in idKC cell cycle and proliferation, where coating with both fibronectin and fibrin demonstrated an increased capacity to augment expression of *CCND1* at 24 h, with no differences between 2D and 3D. Similarly, the expression of *EGFR* and *IL1B* (IL-1β) in idKCs demonstrated minimal differences between 2D and 3D ASC-CM treatment, with only ASC-CM treatment from 3D-Col resulting in a significant decrease, relative to 3D-Fn, in *IL1B* expression. However, expression of *EGFR* experienced a decreasing trend in all 3D groups, whereas both 2D and 3D groups exhibited increasing expression of *IL1B*, relative to baseline idKC expression. Additionally, all 3D ASC-CM samples demonstrated the capacity to significantly decrease expression of p16^ink4a^ (*CDKN2A*), a marker for senescence, relative to their 2D ASC-CM counterparts and the baseline expression in idKCs. Lastly, 3D-Col and 3D-Fn ASC-CM both significantly increased expression of the suprabasal barrier marker filaggrin (*FLG*) in idKCs, whereas only ASC-CM from 3D-Col and 3D-Fib increased expression of the migratory marker *TWIST1* in idKCs (Figure 3D).

Interestingly, in healthy KC populations performed in parallel, 3D-Fib has a more significant effect on KC metabolic and proliferative activity. However, 3D-Col was still the only group to significantly enhance KC migratory activity greater than the baseline control KCs treated with KC-GM (Supplementary Figure S1). Of note, 2D-NC groups for both KC and idKC groups exhibited a decline in migratory activity, relative to baseline control (Supplementary Figure S1).

### 3.4. Augmenting the idKC epidermal phenotype via matrix-dependent modulation of ASC secretome

**Figure 4:**
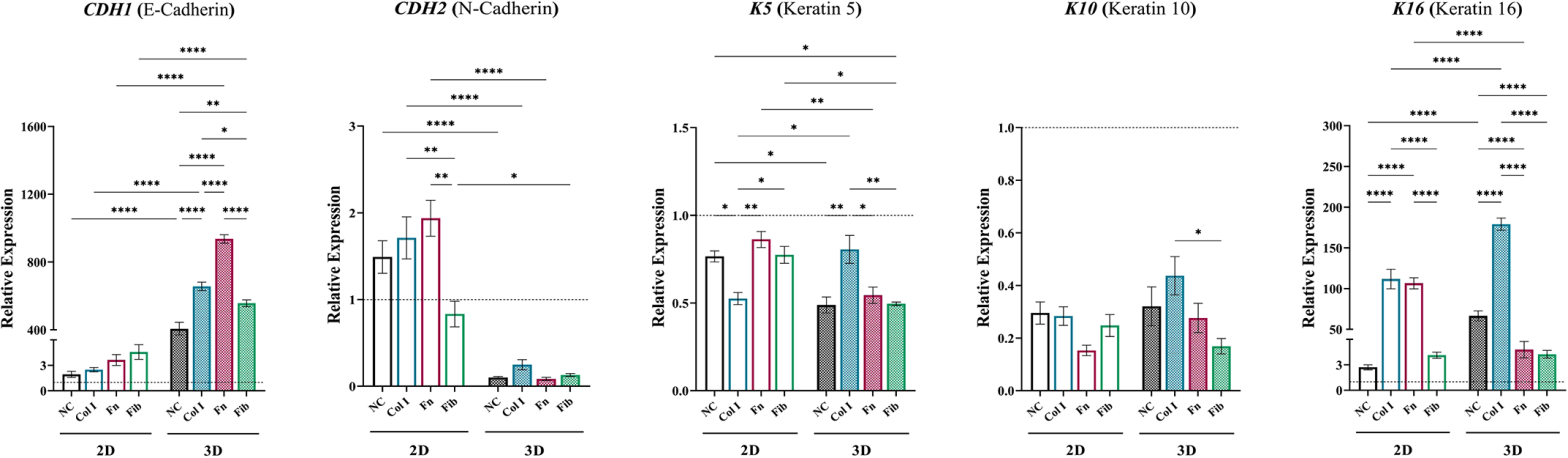
Augmenting the idKC epidermal phenotype *via* matrix-dependent modulation of ASC secretome. qRT-PCR analysis of *CDH1* (E-cadherin), *CDH2* (N-cadherin), *K5*, *K10*, and *K16*. *GAPDH* was used as an internal control. Values are represented as relative fold change to baseline control idKCs indicated by dashed line, using *the* ΔΔCt method. NC, non-coated; Col 1, collagen type I; Fn, fibronectin; Fib, fibrin. Significance denoted as *p < 0.05, **p < 0.01, ***p < 0.001, and ****p < 0.0001.

Assessment of idKC cytokeratins and cell junctional proteins were evaluated to determine changes in the epidermal phenotype of idKCs after treatment with ASC-CM via changes in gene expression with qRT-PCR (Figure 4). Notably, was the significant enhancement in expression of E-Cadherin (*CDH1*) in idKCs treated with all 3D ASC-CM groups, with 3D-Fn providing the most significant stimulus. All 3D coated groups provided variable degrees of enhancement, whereas 2D coated samples provided no significant differences between coating groups. Conversely, relative to control, N-Cadherin (*CDH2*) was significantly decreased in all 3D ASC-CM treatment groups, whereas 2D ASC-CM groups had no significant effect on idKC expression of *CDH2*, relative to baseline idKCs*.* Interestingly, relative to the other coatings, 2D-Col ASC-CM resulted in decreased expression of K5 in idKCs but 3D-Col resulted in an increased expression. The migratory and wound responsive marker K16 was significantly enhanced in idKCs treated with ASC-CM from 2D-Col and 3D-Col, however, 3D-Col resulted in the most significant increase amongst all groups in 2D or 3D. Whereas ASC-CM from 3D-Fn and 3D-Fib resulted in a significant decrease, relative to 3D-NC. The suprabasal differentiation marker K10 exhibited minimal differences between 2D and 3D ASC-CM treatment and between different coatings, although all groups exhibited a decreased expression relative to baseline idKCs.

### 3.5. Matrix-coating within tissue-mimetic system enhances the relative secretion of regenerative compounds from ASCs

**Figure 5:**
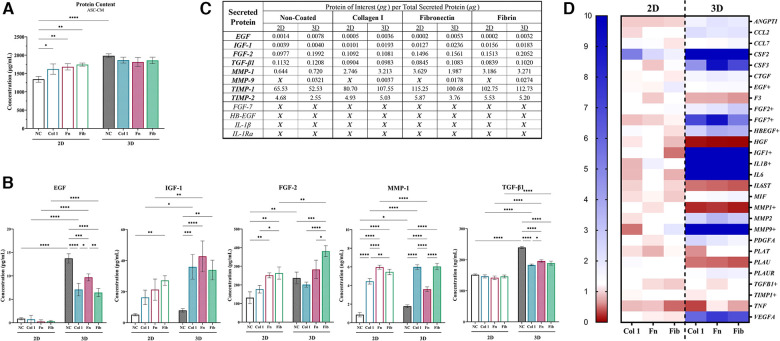
Matrix-coating within tissue-mimetic system enhances the relative secretion of regenerative compounds from ASCs. (**A**) ASC-Cm collected from each group was evaluated for total protein concentration via QuickDrop, BCA, and bradford (coomassie). The figure depicts QuickDrop data. (**B**) A total of twelve (12) ELISAs were performed on ASC-CM samples. Only seven (7) contained a high enough protein concentration above the limit of detection for the ELISA. Five (5) key factors of those seven (7) are depicted in the figure. (**C**) A table to depict the relative concentration of each protein tested (in “*pg*”) in relation to the total amount of secreted protein (in “µ*g”*). An “X” depicts that the sample was below the limit of detection for the ELISA. (**D**) ASCs were evaluated for changes in gene expression of key markers (*28 selected*) *via* a Wound Healing qRT-PCR array (*84 total target*) and depicted with a heatmap. A fold change of ≥10 is denoted as the highest increase in fold change (*dark blue*). The markers selected are associated with secretory activity from ASCs and several align with the proteins of interest for the ELISAs. *GAPDH*, *ACTB*, and *B2M* were the endogenous control genes utilized by the array. NC, non-coated; Col 1, collagen type I; Fn, fibronectin; Fib, fibrin. Significance denoted as *p < 0.05, **p < 0.01, ***p < 0.001, and ****p < 0.0001.

Total protein content within ASC-CM for each group was analyzed, with 2D-NC (1,341 µg/ml) exhibiting significantly less protein within the ASC-CM than all other groups and 3D-NC exhibiting the highest (1,982 µg/ml). Culture within 3D-NC significantly enhanced the overall secretory activity of ASCs, with a ∼48% increase secretion of proteinaceous compounds, similar to previously reported data (50). Interestingly, coating within 2D groups increased the secreted protein concentration to the same level of all other 3D groups, whereas 3D ASC-CM exhibited no difference between coated and non-coated groups (Figure 5A). Subsequently, the concentration of specific secreted protein markers from ASCs that are known to modulate epidermal regeneration were quantified via ELISAs (Figures 5B,C) and ASC gene expression of specific secreted factors was validated (Figure 5D and Supplementary Figure S2). Coating in 2D groups had no effect on modulating the secretion of EGF and TGF-β1, whereas coating in 2D was able to modulate the secretion of IGF-1, FGF-2, and MMP-1, 2D-Fb and 2D-Fn significantly increased the secretion of several factors. 3D culture significantly enhanced the secretion of EGF, IGF-1, FGF-2, MMP-1, MMP-9, TGF-β1, and TIMP-1, relative to 2D groups. 3D-Fib had the most significant effect on secretion of FGF-2. Additionally, all coatings significantly decreased secretion of EGF and TGF-β1 in 3D, whereas all coatings significantly increased secretion of IGF-1 and MMP-1 in 3D, relative to 3D-NC (Figure 5B and Supplementary Figure S3). Interestingly, 3D culture resulted in a significant decline in secretion of MMP-1 in 3D-Fn but a significant increase in the 3D-Col group, relative to their 2D counterparts. Lastly, coating in both 2D and 3D significantly increased secretion of TIMP-1, with a similar pattern in TIMP-1 and MMP-1 secretion (Figure 5B and Supplementary Figure S3). Protein secretion from ASCs was further validated via evaluation of gene expression with a wound healing array. A heatmap demonstrates increased ASC expression of several secretory proteins in 3D relative to 2D groups, with coating in 2D having minimal effects on ASC activity (Figure 5D). A full list of relative gene expressions for the entire array can be found in Supplementary Table S2.

### 3.6. ASC exposed to collagen type I in 3D enhance epidermal regeneration of idKCs via secreted EVs

**Figure 6:**
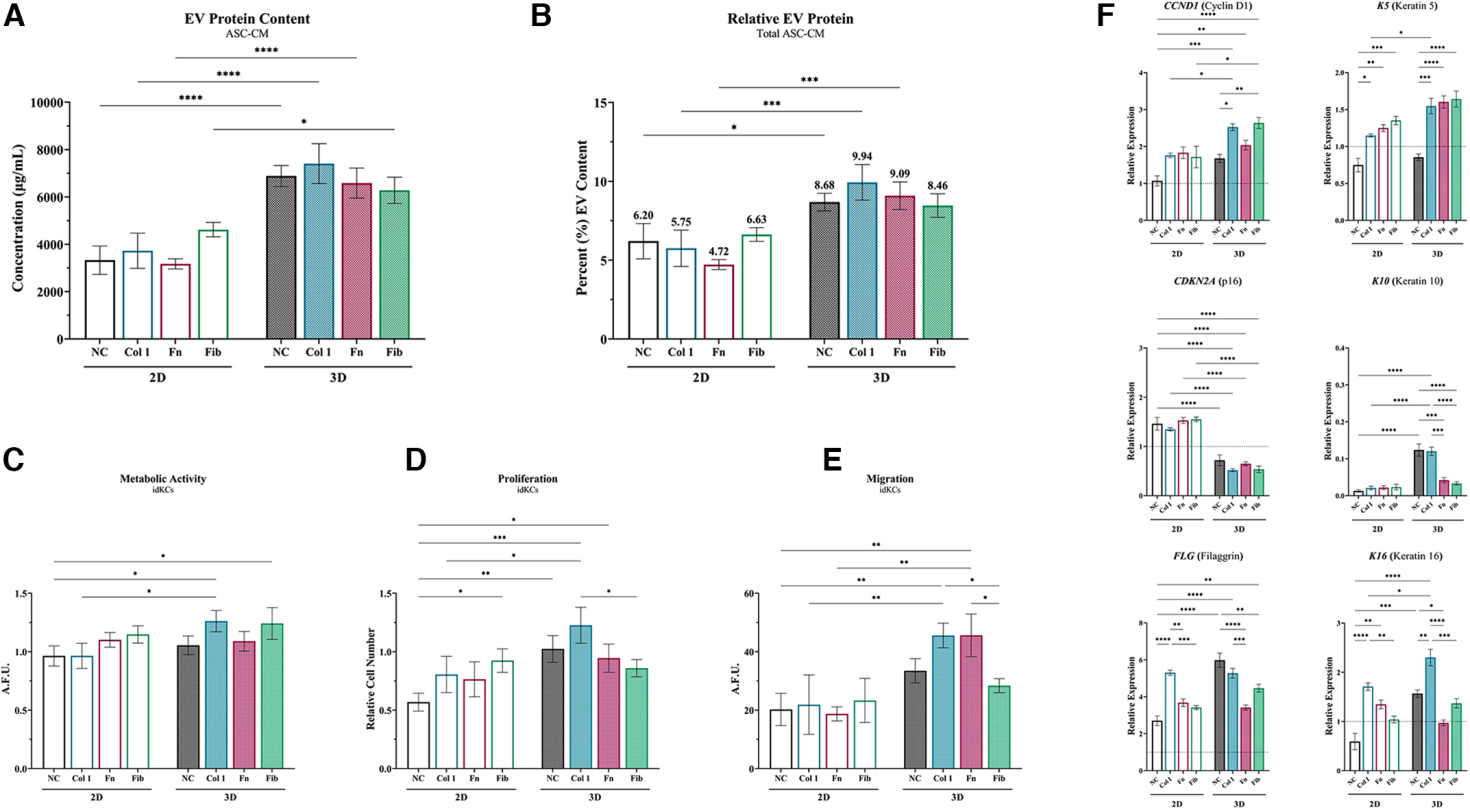
ASC exposed to collagen type I in 3D enhance epidermal regeneration of idKCs *via* secreted EVs. (**A**) ASC-EVs were isolated from 2D (*silhouette*) and 3D (*patterned*) ASC-CM and the relative concentration of EVs per sample were analyzed via protein content. (**B**) The relative quantity of EVs to total secreted protein was then calculated to determine relative compositional changes for each group. (**C**) Metabolic, (**D**) proliferative, (**E**) and migratory activity were evaluated for idKCs treated with KC-GM dosed with 150 µg/ml of EVs. (**F**) qRT-PCR analysis of *CCND1*, *CDKN2A*, *FLG*, *K5*, *K10*, and *K16* was then performed to assess for expressional changes in idKCs. *GAPDH* was used as an internal control. Values are represented as relative fold change to baseline control idKC expression, indicated by dashed line, using the ΔΔCt method. NC, non-coated; Col 1, collagen type I; Fn, fibronectin; Fib, fibrin. Significance denoted as *p < 0.05, **p < 0.01, ***p < 0.001, and ****p < 0.0001.

ASC-EVs from each ASC-CM group were then evaluated for their ability to directly modulate idKC epidermal regeneration activity to demonstrate the relative compositional and quality changes of the different EV populations. Overall, ASCs within the tissue-mimetic 3D system demonstrated enhanced secretion of extracellular vesicles (EVs) for all non-coated and coated samples (∼1.89-fold increase in 3D), relative to each 2D counterpart. However, substrate coating in 2D and 3D did not alter the relative concentration of EVs within ASC-CM for each respective group (i.e., intragroup comparisons) (Figure 6A). Moreover, when controlling for total protein secreted within ASC-CM, the relative composition of EV Protein-to-Total Secreted Protein was significantly greater in 3D ASC-CM groups, relative to their 2D counterparts, except for fibrin (Figure 6B). Analysis of the size distribution of the EVs was performed via NTA and demonstrated that >95% of all particles analyzed were within the 25–250 nm range and ∼2–3× greater particles in 3D relative to 2D, similar to the EV protein quantification data (Supplementary Figure S5).

To assess the relative role of ASC-EVs on the previous benefits of ASC-CM treatment, idKCs were treated with KC-GM media dosed at a concentration of 150 ug/ml of isolated EVs from each respective 2D and 3D group. The idKCs were then evaluated for epidermal functional activity via assessment of metabolic (Figure 6C), proliferative (Figure 6D), and migratory activity (Figure 6E). Notably, EVs from 3D-Col were the only 3D-EVs to result in a significant enhancement in all three idKC functions (metabolic, proliferative, and migratory activity), when comparing each respective 3D coating group to their 2D counterpart (Figures 6C–E). More specifically, 3D-Col EVs resulted in the highest increase in idKC proliferative activity, with ∼2-fold increase relative to 2D-Col (Figure 6D), whereas both 3D-Col and 3D-Fn EVs resulted in a significant increase in idKC migration relative to their 2D counterparts (Figure 6E).

Gene expression analysis was then performed on idKCs after 24 h of ASC-EV treatment (relative to control idKCs) to further validate idKC functional and phenotypic changes (Figure 6F). Coating in 2D was able to result in production of ASC-EVs that enhanced the expression of K5, K16, and FLG, relative to non-coated groups, with 2D-Col exhibiting significantly greater propensity for increasing expression of K16 and FLG (Figure 6F). However, 3D-NC resulted in a significant improvement in idKC expressional profile for all markers except K5, relative to 2D-NC. Notably, 3D coating demonstrated the capacity to modulate ASC-EV activity and improve expression of several idKC markers and resulted in a significant decrease in expression of CDKN2A, relative to all 2D-EV groups. Additionally, 3D-Col was the most consistent group to significantly impact idKC gene expression and was the only 3D-EV group to significantly enhance the expression of all three keratins, K5, K10, and K16, relative to their 2D counterpart (Figure 6F).

## 4. Discussion

Incomplete and failure of wounds to “close” efficiently due to trauma, surgery, acute or chronic disease, and radiation-induced tissue damage often results in the progression of chronic, non-healing wounds and affects millions of people every year (53). Notably, diabetic wounds are among the most prevalent of all chronic wounds and currently affect ∼15% of all diabetics in the US. With the aging population and rise in diabetes, the prevalence of chronic diabetic wounds is expected to increase (54). Thus, therapies tailored for diabetic wounds are a highly researched topic. However, currently there is minimal research investigating therapies for improving the epidermal regeneration capacity within diabetic wounds specifically. Therefore, there remains a critical need to develop a therapy that can circumvent the loss of proper keratinocyte functionality and restores the appropriate epidermal regeneration capacity of wounds to prevent further progression of chronic diabetic wounds.

Early studies with MSC populations in *in vitro* and *in vivo* wound models have demonstrated the ability to promote pro-wound healing activity in a number of key wound healing cells populations, and modulate the epidermal regeneration activity of wounds (30). Moreover, *in vivo* animal models and clinical trials with diabetic wound models have shown the ability for ASCs to directly augment wound healing, enhance the rate of wound “closure” and improve overall outcomes (30, 34). The injection of ASC and ASC-loaded wound dressings for treatment of diabetic ulcers have demonstrated promise. However, retainment of ASCs within the wound site via utilization of a wound dressing or other delivery vehicle appears to be critical for enhancing therapeutic benefits with ASC therapies (55, 56). This is thought to be, in part, a result of the proximity of the “wounded tissue stimulus” to ASCs, rather than the radial diffusion and cell death that is commonly seen in injected cell therapies, which ultimately allows the ASCs to continuously adapt/respond to the evolving wound tissue environment.

Although promising, cell-based therapies do have their limitations and can be highly variable depending on the baseline viability and robustness of the ASC source, which is often compromised in patients exhibiting chronic wounds. Recent investigations into the adaptive and secretory nature of ASC populations to priming stimuli have provided a unique opportunity for developing regenerative wound therapies that are capable of circumventing many of the limitations of autologous cell therapies (57). It is conceivable that the regenerative capacity seen from prior ASC therapies are, in part, due to the secretion of various paracrine compounds from ASCs that contain anti-oxidants, anti-inflammatory, anti-proteolytic, and targeted EV compounds. However, to date, most therapies are investigating the utilization of 2D cultured MSC populations, which is known to result in decreased viability, terminal differentiation, and a loss of regenerative capabilities, which drastically hinders the translatability of potential MSC-derived regenerative therapies (58–60).

Based on prior studies that have demonstrated the ability for 2D cultured ASCs to enhance wound healing activity, in this study, a unique tissue-mimetic 3D system was utilized and compared to a traditional 2D system. The 3D system is mechanically similar to native adipose tissue and thus more physiological for ASC populations. Therefore, this study uniquely investigated the effect of multiple wound matrix coatings on the ASC secretome functionality while controlling for the mechanical input from 2D and 3D. The utilization of different matrix-derived proteins used as priming stimuli in both 2D and 3D systems directly assessed for the role of the wound matrix in altering ASC secretory activity and subsequent modulation of the epidermal regeneration capacity of the ASC secretome. Based on prior studies that have demonstrated how this tissue-mimetic system helps maintain a more regenerative and secretory ASC population (47, 50), our hypothesis was that a more robust ASC population will be more responsive to priming stimuli and secrete higher concentration of specific pro-regenerative factors accordingly, relative to 2D culture. This was supported in this study multiple ways, including the increased secretion of soluble protein (Figure 5A), EVs (Figure 6A), and anti-oxidants (Supplementary Figure S4) by ASCs within the 3D system, relative to 2D. Priming within the 3D system consequently led to greater diversification of the ASC secretome functionality and composition, compared to the 2D secretome which saw less overall diversity between different coatings.

The matrix coatings within this study were selected based on the following principles. Fibrin has a native role in wound healing and has been shown to improve wound outcomes *in vivo*. Fibrin has demonstrated the ability to further enhance wound healing outcomes of MSC-based treatments *in vivo*, including complex diabetic wounds and has been shown to promote the secretion of several specific soluble factors, including VEGF, EGF, and FGF (29, 61, 62). Fibronectin is a critical component to the extracellular matrix and basement membrane, directly interacts with collagen during wound healing, and exhibits high turnover during cutaneous wound healing due to frequent damage to the basement membrane (63, 64). Lastly, collagen type I is the most abundant matrix protein in tissue, including skin, and plays a key role in wound healing and epidermal regeneration. Notably, collagen type I makes up a substantial portion of the dermal collagen that becomes exposed upon wounding, which is considered to provide a key migratory stimulus for wound healing cells (65–67). Moreover, a number of studies have investigated the benefit of encapsulating MSC populations in collagen type I and have demonstrated improved wound healing outcomes (65, 68). Thus, these three matrix proteins were chosen because they provided a diverse array of substrates involved with different components of native wound healing. Future studies will investigate the role of other substrates as well as heterogenous mixtures of substrates paired with additional environmental or biochemical stimuli (e.g., hypoxia) as a means to generate a tissue-mimetic *in vitro* system capable of generating tailored compositions of factors for specific wound applications.

Previous studies have shown that hyperglycemia in native diabetic patients can induce a phenotypic switch in KCs altering their barrier functionality, while also inhibiting proper migration and proliferation activity (69, 70). Additionally, studies show that a “functional” diabetic KC phenotype can be induced *in vitro* by high-dose glucose exposure to KCs, with similar cellular changes seen in KCs from a patient with diabetic wounds, including decreased proliferation and migration (Figure 2) (51, 52, 69, 71). An inducible system was utilized in this study in order to allow for a non-diabetic control KC population to be performed in parallel. Whereas primary KCs from a diabetic patient neither guarantees functional deficits in wound healing nor allows for a non-diabetic control from the same patient.

ASC-CM assays with idKCs revealed that coating with collagen type I within the tissue-mimetic system enhanced the epidermal regeneration functionality of the ASC secretome more significantly than fibrin or fibronectin, for both 2D and 3D (Figures 3A–C). Interestingly, ASC-CM from 3D-Fib provided a stronger metabolic and proliferative stimulus to healthy KCs, rather than 3D-Col (Figure 1). These data provides new insight and indicates a possible shift in sensitivity of what factors may be necessary to achieve epidermal regeneration in healthy vs. diabetic KCs, further highlighting the importance of tailored therapies for specific wound applications. The four highest increased differentiation markers of 3D ASC-CM treated idKCs were CDH2, FLG, TWIST1, and K16, all of which are critical responsive genes for epidermal regeneration. Of note, ASC-CM from 3D-Col and 3D-Fn both resulted in the two highest upregulations of filaggrin and e-cadherin, key proteins involved in barrier functionality within the suprabasal epidermis, as well as cell sheet formation (Figure 3D). However, only 3D-Col ASC-CM resulted in a significant increase in expression of the basal marker (keratin 5), the suprabasal differentiation marker (keratin 10), and migratory and wound responsive marker (keratin 16) in idKCs, relative to other coatings (Figure 4A–C). 2D-Col was only able to promote increased K16 expression, to a lesser extent, relative to the other 2D coatings. Together these data suggest that ASC-CM from 3D-Col had the highest capacity overall for augmenting a diverse and regenerative KC phenotype that may be associated with the improved barrier formation and functional activity seen in idKCs, and was the only group to significantly enhance the migratory capacity of idKCs relative to their baseline state (Figure 3C).

ASC-CM from all 3D groups did demonstrate increased production of key signaling factors, relative to their 2D counterparts. Interestingly, although 3D-Col resulted in the greatest capacity to augment idKC functional activity, 3D-Col did not result in the highest secretion of any key soluble proteins that are considered to be critical for epidermal regeneration functionality, including EGF, IGF-1, FGF-2, or FGF-7 (KGF). This could suggest a few things; that the relative composition and stoichiometric balance between several factors may be important, idKCs may rely on other proteins not tested, or non-proteinaceous factors may play a more significant role in idKC regenerative activity, such as EVs. Notably, the concentration of EVs secreted by ASCs was increased for each coating group in 3D relative to their 2D counterparts, but no difference was noted between 3D groups. This suggests the increased production of EVs was likely dependent on the tissue-mimetic environment and not the coatings. These data further supports the concept that ASCs maintain a greater propensity for regenerative secretory activity in 3D, whereas exogenous factors and/or coating substrates can be used to fine-tune and tailor the relative composition of the secretome.

To test whether the EV fraction was driving any of the functional changes seen in the idKCs, a study was performed where KC-GM was dosed with 150 µg/ml with EVs to allow for evaluation of the functional quality of the EVs on an EV-to-EV basis. Upon controlling for total amount of EVs, idKCs exhibited varying functional changes after ASC-EV treatment within 24 h. Both idKC metabolic and proliferative activities were significantly enhanced with 3D-Col relative to other ASC-EV groups. Additionally, both 3D-Col and 3D-Fn significantly increased the migratory capacity of idKC. Therefore, 3D-Col was the only 3D coating to augment idKC functional activity across all three assays, relative to their 2D counterparts.

Interestingly, the effect of ASC-EV treatment on idKC metabolic and proliferative activity exhibited a similar pattern as the data from “Full/Complete” ASC-CM treatment in Figure 3. Suggesting that idKCs metabolic and proliferative activity may be more significantly impacted by EV entities rather than secreted proteinaceous factors. Conversely, 3D-Fn did not have a significant impact on idKC migratory activity originally when using the “Full/Complete” ASC-CM in Figure 3. Although the migratory activity was significantly enhanced with 3D-EVs, the relative extent of migration in 24 h was less with only ASV-EV (3D-Col was ∼44%) treatment when compared to the “Full/Complete” ASC-CM (3D-Col was∼75%). This suggests that there is likely an additive or synergistic effect between ASC-EVs and non-EV soluble factors in augmenting idKC migratory capacity. Together these data reveal that the quality/composition of contents within the EVs varies in a substrate-dependent manner, with 3D-Col derived EVs having the highest propensity to consistently augment epidermal regenerative functionality in idKCs.

The effect of EV dosing on idKC gene expression also demonstrated that 3D-EVs were able to significantly modulate idKC expressional patterns to a greater extent than 2D-EVs. For example, *CCND1* and *CDKN2A* expression in idKCs was significantly altered after treatment with 2D and 3D EVs. Therefore, ASC-EVs may play a key role in modulating the relative progression of the idKC cell cycle, demonstrated by increased expression of the proliferation marker *CCND1* after 3D-Col and 3D-Fib EV treatment, as well as how all 3D-EVs significantly reduced the senescence marker *CDKN2A*. The significant decline in *CDKN2A* relative to control idKC expression suggests that ASCs in a tissue-mimetic system may be secreting EVs containing senescence-protective compounds. Next, the suprabasal differentiation marker, *FLG*, exhibited similar expression patterns between ASC-EVs from the 2D and 3D coated groups, whereas the 3D-NC resulted in a significant increase in expression relative to 2D-NC. Thus, the tissue-mimetic properties of the 3D system are likely broadly beneficial in modulating FLG expression, but the benefits of 3D system are negated by the addition of matrix-derived compounds which appear to have a stronger impact on EV functionality towards altering *FLG*, demonstrated by the similar idKC expression profiles after treatment with either 2D or 3D EVs from coated samples. Lastly, ASC-EV were able to modulate the expression of idKC keratins, with 3D-Col being the only 3D coating group to significantly increase all three keratin markers relative to the respective 2D counterparts. This included the key “wound responsive” keratin, K16. These data were similar to the expression analysis performed on “Full/Complete” ASC-CM in Figure 4, where 3D-Col was superior to all other groups in promoting K16 expression. The superior idKC functionality after treatment with 3D-Col EVs may be related to the capacity to modulate K16 activity in idKCs, but will require further investigations.

The EV data within this study potentially suggest that KCs may become more dependent on signaling factors found within EVs when assuming a diabetic-like phenotype. To the authors' knowledge, this is the first demonstration of diverging effects of ASC-derived EVs based on substrate coatings and the potential shift in sensitivity between external signaling molecules when KCs assume a diabetic-like phenotype. The dynamic mechanisms at play require further extensive investigations, although one possible explanation could be that the signaling dynamics shifts towards favoring uptake of EV-like particles in diabetic KCs as a result of a change in cell surface receptors. This could potentially be due to cellular and protein damage caused by super-physiological glucose levels and increased oxidative activity, thus the sensitivity of diabetic KCs to external signaling factors may be altered. Additionally, as we can see from the ASC secretome effects from the 2D system, there is much less heterogeneity in functional effects on the idKCs relative to the coated 3D ASC secretome. This indicates that not only is there a coating/material effect but likely also a combinatorial effect with the 3D hydrogel system on ASC phenotype and functionality. Moreover, the coating effect on the ASC secretome may be further augmented because the ASCs in 3D are in a more tissue-mimetic environment and, thus, are retaining their inherent adaptive nature and able to more appropriately respond to the coating stimuli.

There are elements within this study that are limiting in their current form and require further investigations, but do not ultimately detract from the overall findings. ASC populations may exhibit donor-specific variability in their regenerative properties that may alter the secretory activity from one to the next. However, the comparative study design between 2D and 3D, as well as between different coatings, allowed for any donor specific effects to impact each group to the same extent. Similarly, the effects of the ASC-CM were only tested in an “induced-diabetic” KC model. Although this allowed for healthy KCs to be tested in parallel, there are likely still differences in KC populations isolated from chronic diabetic wounds at different stages of the chronic wound progression. Additionally, future studies with other wound healing cell populations should be performed to establish a more holistic idea as to the role of ASC secretome components on diabetic wound healing. Although the utilization of only *in vitro* analyses can also be limiting, the utilization of a wide-range of functional, proteomic, and RNA assays provided a holistic perspective that provided data that can be used for a future *in vivo* study designs and provide the framework for tailoring the composition of the ASC secretome. Notably, previous studies have already established the similarity between the *in vitro* diabetic model used in this study and the “functional” phenotype of KCs isolated from diabetic wounds. Additional investigations into the dynamic and inter-related role of substrate/coating materials and the X-Block hydrogel system utilized is also warranted. The authors speculate that the ASC phenotype is shifting when exposed to the coating materials due to retainment of their inherent adaptive nature, however, to what extent remains in question and will require follow up studies. Lastly, although outside the scope of this study, future studies into the dynamic array of secretome compounds, including mass spectroscopy of proteins and investigations into the contents within EV (e.g., proteins, ROS, nucleic acids, miRNA), should be further investigated to better understand how certain environmental conditions shift the secretome composition. It is possible that the same key proteinaceous compounds that drive healthy epidermal regeneration still promote epidermal regeneration in diabetic KCs, but are packaged within EVs instead of secreted as soluble proteins, and thus were not detected via the ELISAs.

Within this study, the significance of utilizing a tissue-mimetic environment for cell culture is highlighted and the role of maintaining a more regenerative MSC-like population for development of regenerative therapies is evaluated. More specifically, the ability for the different 3D ASC-CM groups to display varying functional capabilities, whereas 2D ASC-CM groups displayed minimal differences between the different coating groups supports the hypothesis that ASC are able to adapt to environmental stimuli more readily within the tissue-mimetic system. The authors demonstrate the tailorable role that dimensionality, mechanics, and substrate binding have on influencing ASC secretory activity and the subsequent functional changes seen within the ASC secretome. These parameters are only a small part of a larger understanding, but demonstrate that *in vitro* systems looking to control MSC-like populations and their secretome must consider a multitude of parameters to truly understand and control their activity. A variety of other parameters to consider include the incorporation of environmental (e.g., hypoxia), chemical (e.g., growth factors/cytokines), mechanical (e.g., dynamic compression), or biological stimuli (e.g., co-culture), as well as the addition of specific drugs to better understand the role of the secretome in drug response. Thus, this study offers insight into the future development and potential of tailorable acellular byproducts that can be prefabricated for a multitude of soft tissue wound healing applications, as well as providing the opportunity to more effectively evaluate native cell responses to a variety of stimuli.

## 5. Conclusion

Ultimately, previous studies have demonstrated that ASCs can be primed with exposure to different compounds and environmental conditions to enhance specific functions *in vivo* (e.g., hypoxia priming to enhance angiogenic activity). However, this study offers insight into the comparative effects of matrix-derived compounds on the ASC secretome functionality in regard to epidermal regeneration of idKCs. The potential benefits of tailored ASC biologics on the epidermal regeneration capacity has yet to be demonstrated. Within this study, the role of ASC-secretome on modulating key epidermal regeneration signaling pathways in idKCs is revealed, including the ability to alter keratin expression, promote suprabasal differentiation, and improve barrier formation. More specifically, the role of ASC-EVs within the ASC secretome was evaluated and shown to be a potential key driver of idKC functional responses, suggesting a dynamic shift in signaling towards favoring EV-like compounds when KCs assume a diabetic-like phenotype. Moreover, this study offers insight into the critical role of tissue-mimetic culture on achieving robust MSC-like populations that retain a more dynamic secretory phenotype and are able to adapt more readily to changes in environmental conditions. Whereas 2D culture resulted in significantly less changes when exposed to different coating substrates. Thus, by introducing predetermined exogenous stimuli within a tissue-mimetic system, the secretory product composition can be tailored and fine-tuned, providing an opportunity to generate an array of functional diverse biologics for different applications.

## Data Availability

The original contributions presented in the study are included in the article/**Supplementary Material**, further inquiries can be directed to the corresponding author/s.
