# Supplementary material for: Tailoring the secretome composition of mesenchymal stem cells to augment specific functions of epidermal regeneration: an in vitro diabetic model
Source: Front Med Technol. 2023 Jun 12;5:1194314. doi: 10.3389/fmedt.2023.1194314 (PMC10291509; doi:10.3389/fmedt.2023.1194314)
Supplement: Supplementary file 1 [file Datasheet1.docx]

**Supplemental Data and Figures**

**
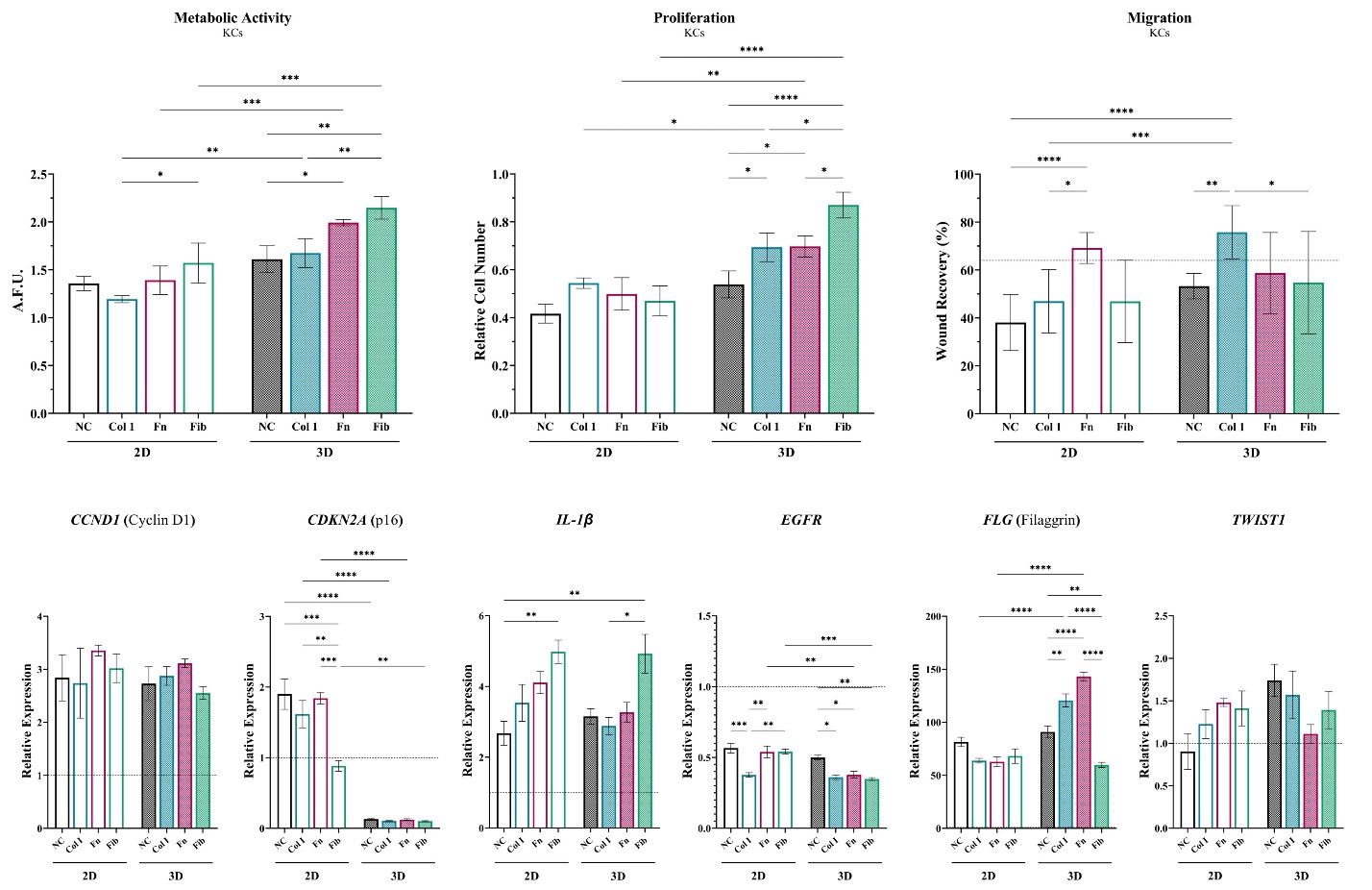
**

**Supplemental Figure 1: Matrix Substrates Alter ASC Secretion of Factors that Modulate Epidermal Regeneration Functional Activity in KCs.** The effect of ASC-CM from 2D (*silhouette*) and 3D (*patterned*) systems that were coated (or non-coated control) was evaluated for ability to modulate “healthy” KC metabolic, proliferative, and migratory activity (*Top row*), was done in parallel to idKCs. Functional activity data are denoted as relative change to baseline control idKCs, which were cultured with keratinocyte growth media (KC-GM). Migratory data depicted as percent (%) area recovered. Dashed line depicts idKCs control. (*Bottom row*) qRT-PCR analysis of *CCND1*, *CDKN2A*, *IL1B*, *EGFR*, *FLG*, and *TWIST1*. *GAPDH* was used as an internal control. Values are represented as relative fold change to baseline control idKC expression, indicated by dashed line. NC = non-coated, Col 1 = collagen type I, Fn = fibronectin, Fib = fibrin. Significance denoted as *p < 0.05, **p < 0.01, ***p < 0.001, and ****p < 0.0001.

**
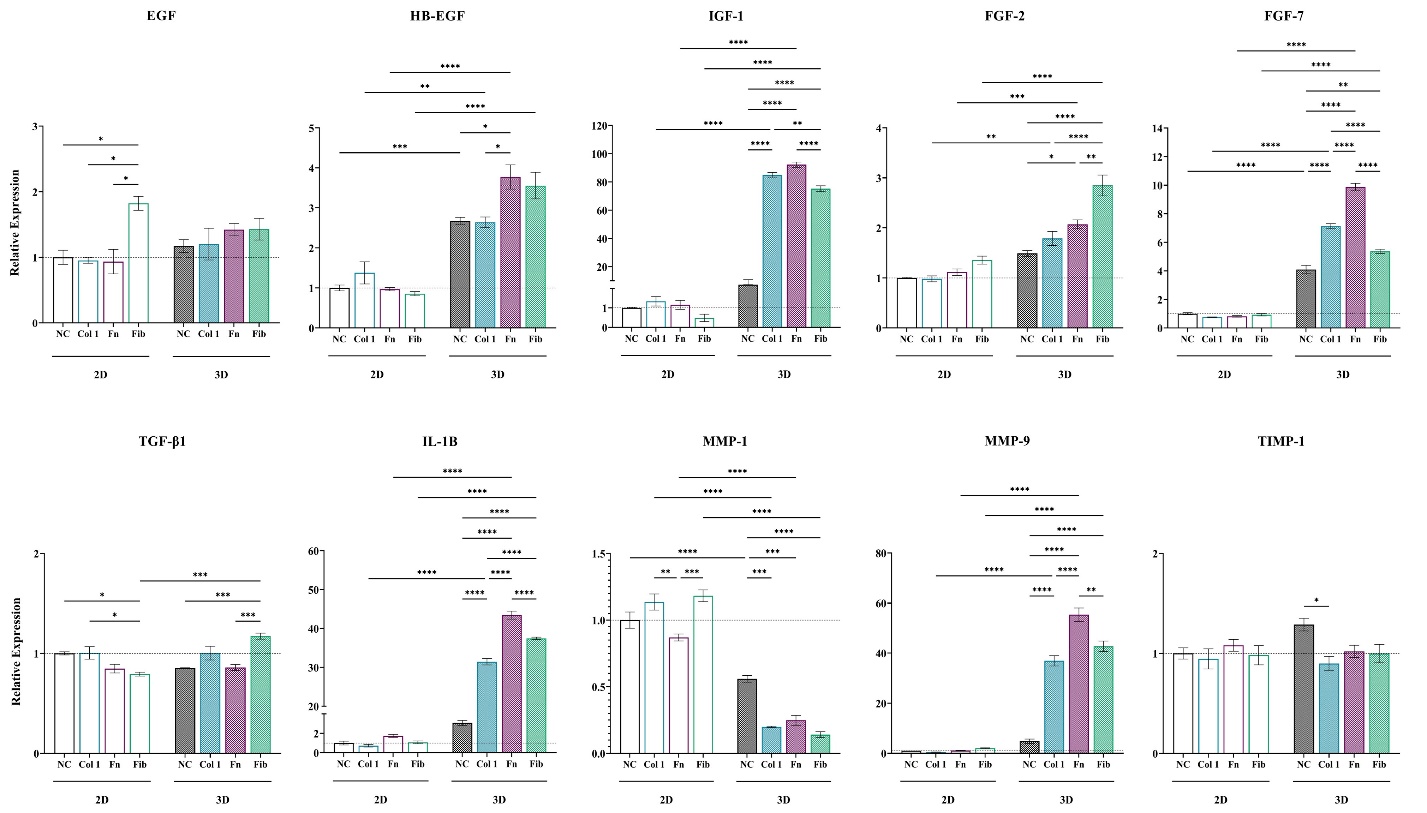
**

**Supplemental Figure 2: ASC Expressional Changes in Key Growth Factors and Cytokines.** The qRT-PCR array data were generated into individual comparative graphs for ten (10) of the key secretory proteins that were also evaluated with ELISAs.

**
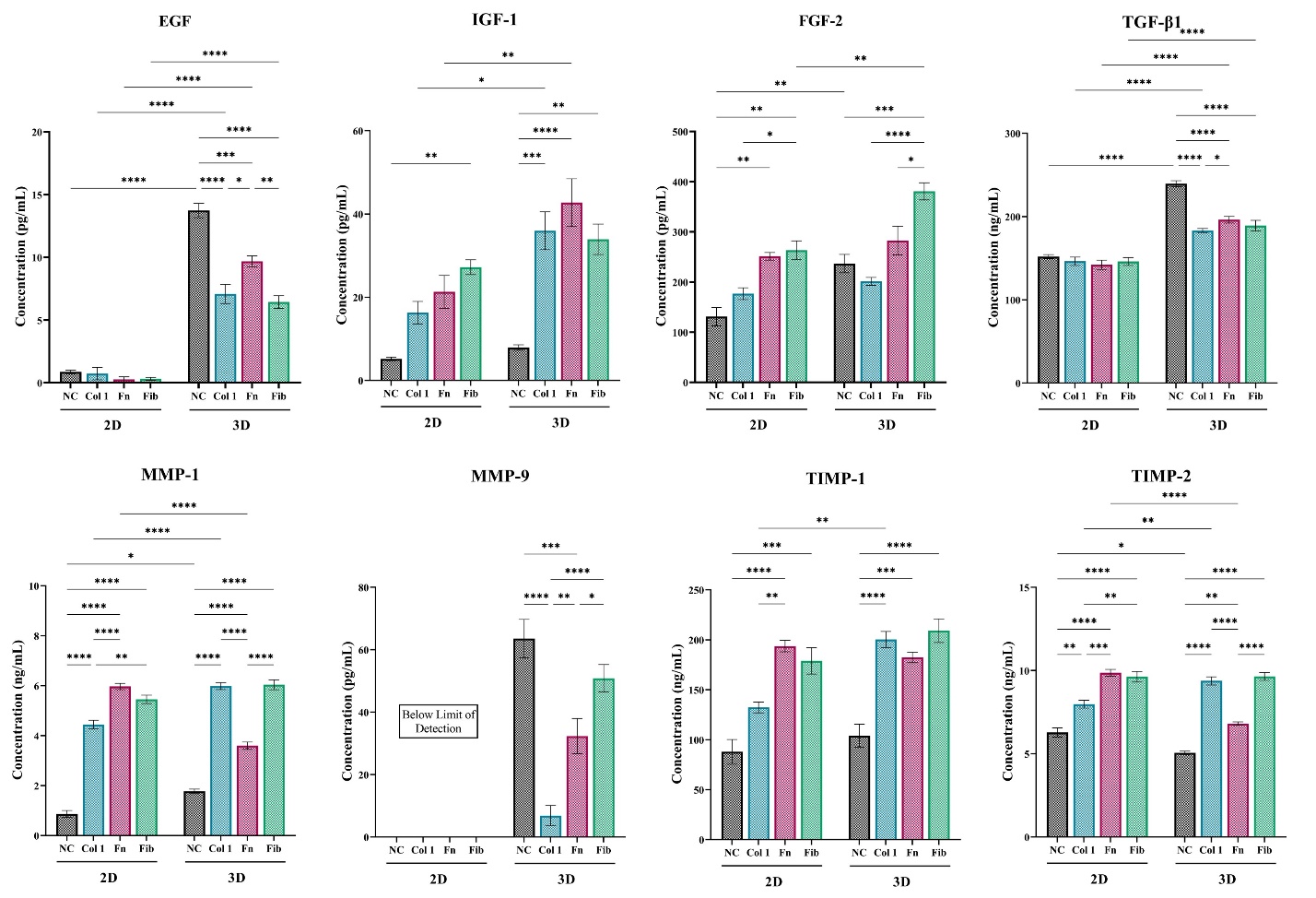
Supplemental Figure 3: ASC-CM Soluble Protein Quantification.** Twelve ELISAs were used to quantify specific protein compounds known to be important for epidermal regeneration natively, including EGF, HB-EGF, IGF-1, FGF-2, FGF-7 (KGF), TGF-β1, MMP-1, MMP-2, TIMP-1, TIMP-2. IL-1β, and IL-1Ra. Of the twelve, only eight had a sample above the limit of detection, seven of which were above for both 2D and 3D. MMP-9 was not detected in 2D (*leftmost columns*) samples but was in 3D (*rightmost colulmns*). NC = non-coated, Col 1 = collagen type I, Fn = fibronectin, Fib = fibrin. Significance denoted as *p < 0.05, **p < 0.01, ***p < 0.001, and ****p < 0.0001.

**
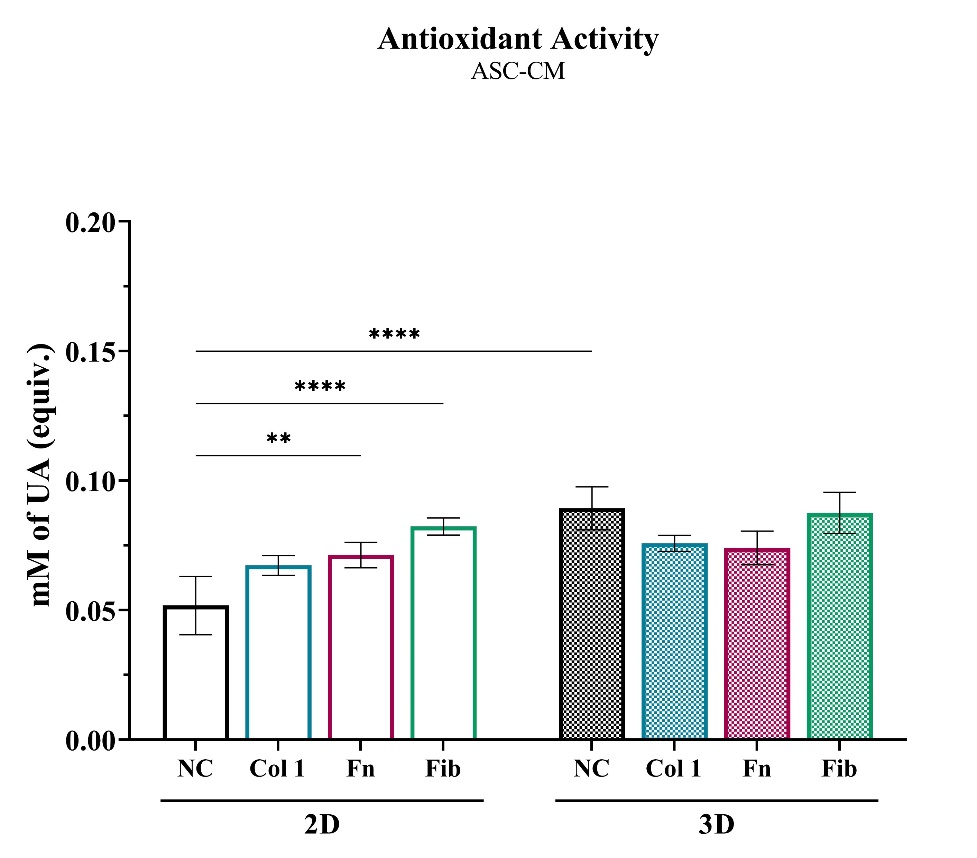
**

**Supplemental Figure 4: Quantification of Relative Antioxidant Activity within ASC-CM**. ASC-CM was collected from 2D (*silhouette*) and 3D (*patterned*) cultured systems, and antioxidant activity was assessed with a Total Antioxidant Capacity (TAC) Assay kit. In short, the reduction of copper (II) to copper (I) by antioxidant activity is assessed, with the naturally occurring antioxidant uric acid, used as a control standard for the kit. Antioxidant activity of ASC-CM was therefore measured in “mM equivalents” of uric acid.

**
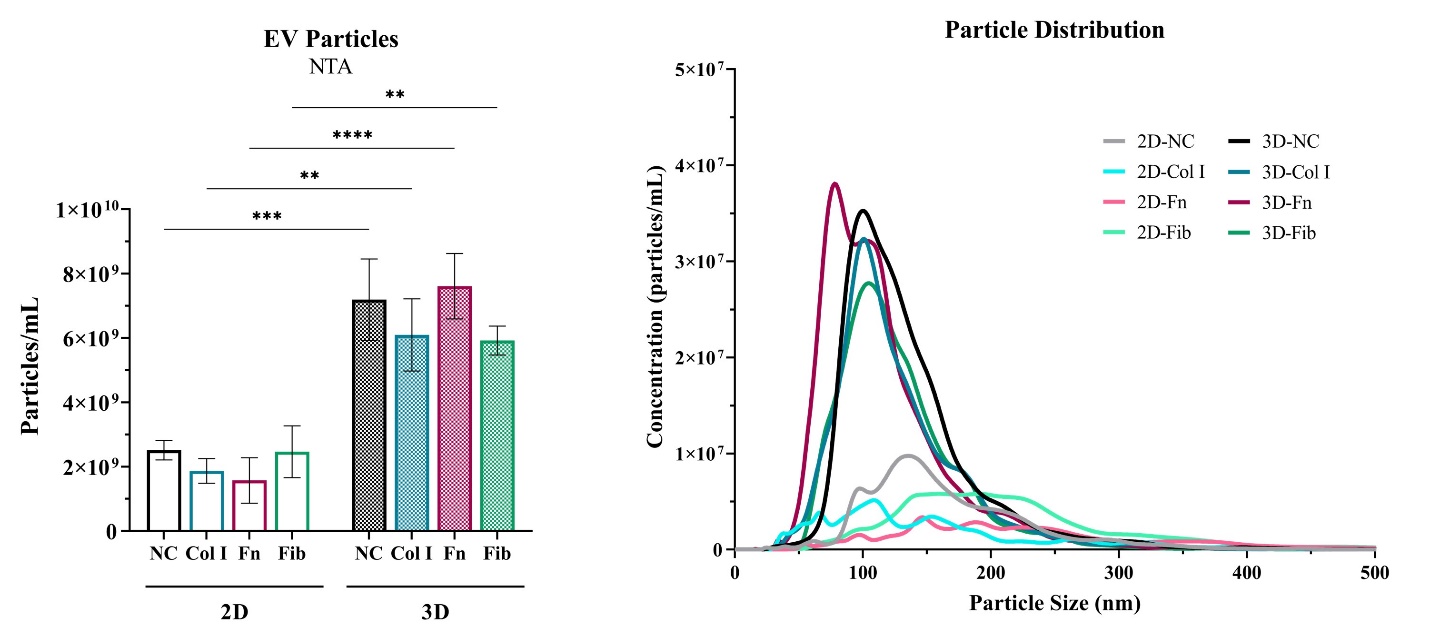
**

**Supplemental Figure 5: Characterization of EV Population within ASC-CM**. The isolated EV fraction was quantified via NTA to establish particle counts for concentration. Significance denoted as *p < 0.05, **p < 0.01, ***p < 0.001, and ****p < 0.0001.


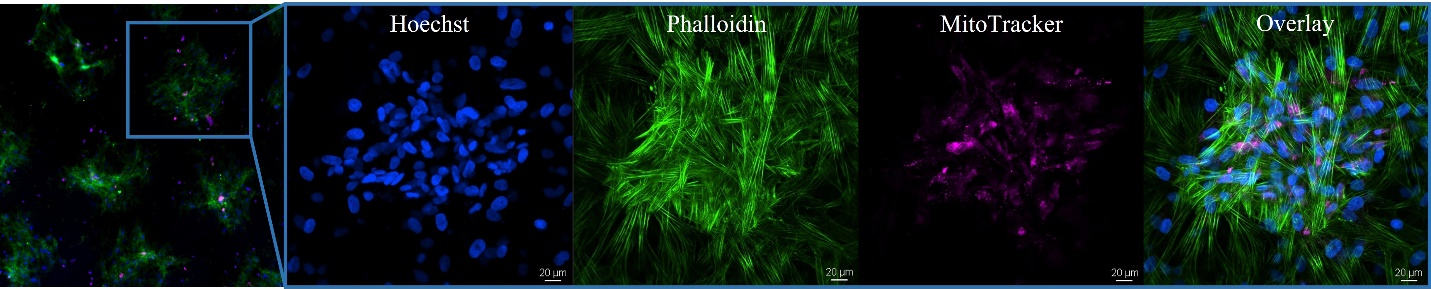


**Supplemental Figure 6: Fluorescent Imaging of ASCs Cultured within the 3D Hydrogel System**. ASCs were seeded and cultured within the 3D Hydrogel system, fixed with 4% PFA, and labeled with Hoechst (*Blue*), Phalloidin (*Green*), or MitoTracker (*Magenta*). Low magnification (4x) of the labeled ASCs within the hydrogel system (*Far Left Panel*) is paired with higher magnification (20x) images that focus on a single pore structure within the hydrogel. Images were acquired by Nikon on their AXR Confocal Imaging System and a z-stack reconstructed was performed.


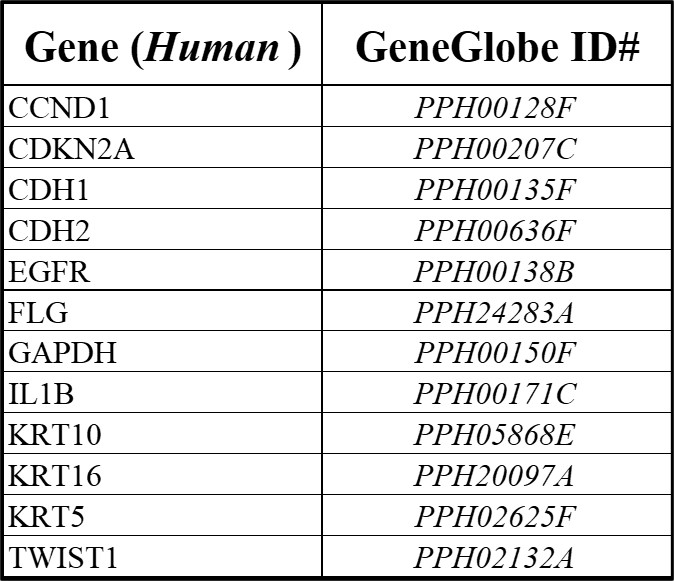


**Supplemental Table 1: GeneGlobe ID Numbers Used for qRT-PCR.** Only human primers were evaluated.


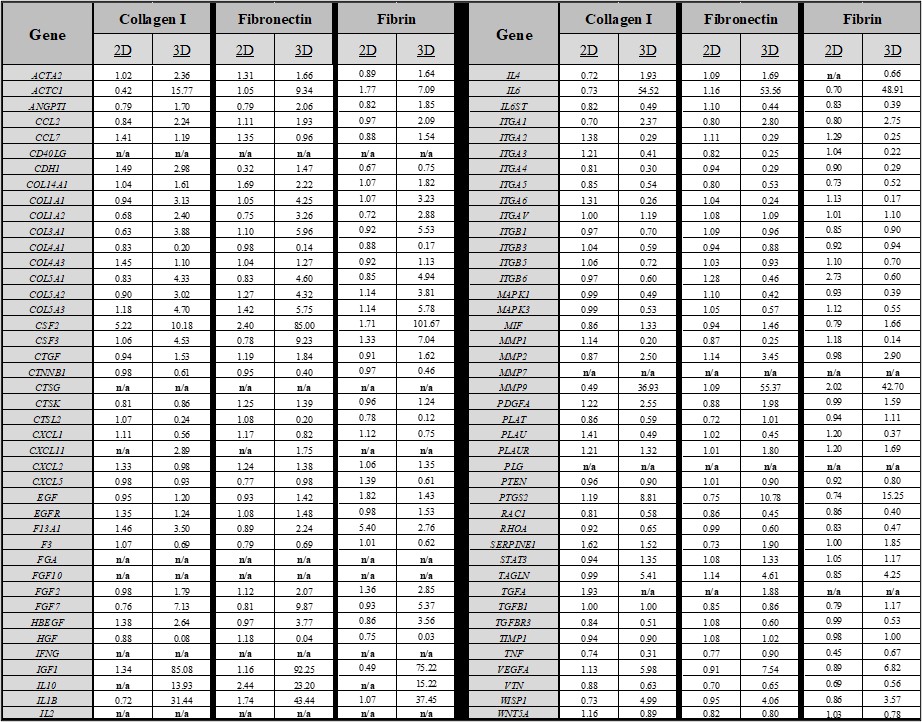


**Supplemental Table 2: ASC Wound Healing Phenotypic Gene Array.** ASCs gene expression evaluated with microarray for 84 targets. Values depict relative fold change to idKC expression. NC = non-coated, Col 1 = collagen type I, Fn = fibronectin, Fib = fibrin.
